# Realistic C-arm to pCT registration for vertebral localization in spine surgery: A hybrid 3D-2D registration framework for intraoperative vertebral pose estimation

**DOI:** 10.1007/s11517-022-02600-5

**Published:** 2022-06-10

**Authors:** Roshan Ramakrishna Naik, Anitha H, Shyamasunder N Bhat, Nishanth Ampar, Raghuraj Kundangar

**Affiliations:** 1 Department of Electronics and Communication Engineering, Manipal Institute of Technology, Manipal Academy of Higher Education, Manipal, Karnataka 576104 India; 2 Department of Orthopaedics, Kasturba Medical College, Manipal Academy of Higher Education, Manipal, Karnataka 576104 India

**Keywords:** Preoperative computed tomography, Intraoperative 3D-2D registration, Intensity-based 3D-2D registration, Coarse registration, Iterative control point registration, C-arm pose modeling

## Abstract

**Abstract:**

Spine surgeries are vulnerable to wrong-level surgeries and postoperative complications because of their complex structure. Unavailability of the 3D intraoperative imaging device, low-contrast intraoperative X-ray images, variable clinical and patient conditions, manual analyses, lack of skilled technicians, and human errors increase the chances of wrong-site or wrong-level surgeries. State of the art work refers 3D-2D image registration systems and other medical image processing techniques to address the complications associated with spine surgeries. Intensity-based 3D-2D image registration systems had been widely practiced across various clinical applications. However, these frameworks are limited to specific clinical conditions such as anatomy, dimension of image correspondence, and imaging modalities. Moreover, there are certain prerequisites for these frameworks to function in clinical application, such as dataset requirement, speed of computation, requirement of high-end system configuration, limited capture range, and multiple local maxima. A simple and effective registration framework was designed with a study objective of vertebral level identification and its pose estimation from intraoperative fluoroscopic images by combining intensity-based and iterative control point (ICP)–based 3D-2D registration. A hierarchical multi-stage registration framework was designed that comprises coarse and finer registration. The coarse registration was performed in two stages, i.e., intensity similarity-based spatial localization and source-to-detector localization based on the intervertebral distance correspondence between vertebral centroids in projected and intraoperative X-ray images. Finally, to speed up target localization in the intraoperative application, based on 3D-2D vertebral centroid correspondence, a rigid ICP-based finer registration was performed. The mean projection distance error (mPDE) measurement and visual similarity between projection image at finer registration point and intraoperative X-ray image and surgeons’ feedback were held accountable for the quality assurance of the designed registration framework. The average mPDE after peak signal to noise ratio (PSNR)–based coarse registration was 20.41mm. After the coarse registration in spatial region and source to detector direction, the average mPDE reduced to 12.18mm. On finer ICP-based registration, the mean mPDE was finally reduced to 0.36 mm. The approximate mean time required for the coarse registration, finer registration, and DRR image generation at the final registration point were 10 s, 15 s, and 1.5 min, respectively. The designed registration framework can act as a supporting tool for vertebral level localization and its pose estimation in an intraoperative environment. The framework was designed with the future perspective of intraoperative target localization and its pose estimation irrespective of the target anatomy.

**Graphical abstract:**

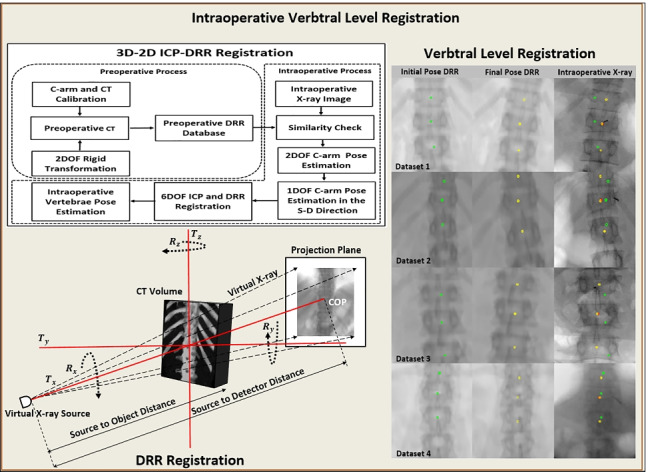

## Introduction

In spinal procedures, most of the local hospitals employ C-arm as an intraoperative imaging system for vertebral level identification, and also for positioning the surgical instruments relative to the tissue anatomy. Spinal surgeries are considered arduous due to varieties in pathological cases, variable vertebrae curvature, and periodic vertebral similarities [1]. In addition, the limited 2D views of spinal X-ray images reduce the perception of positioning pedicle screws relative to vertebral tissue and its vulnerable neighboring tissue regions. Furthermore, arbitrary field of view (FOV) of the target region and low-contrast X-ray images complicate decision-making in an intraoperative environment. Problems addressed above are addressed by accurate identification and localization of target regions in intraoperative images with the support of preoperative images such as computed tomography (CT), magnetic resonance imaging (MRI), or ultrasound (US).

Precisely, in the pedicle screw insertion procedure, once a patient is positioned in the prone position, the vertebral levels are exposed and X-ray images are acquired by positioning C-arm’s X-ray source below the operating table. X-ray images are acquired to identify the right vertebral levels for pedicle screw insertion by placing surgical tools or markers. Multiple X-ray images are acquired to visualize anatomical regions of the fractured vertebrae, to analyze the pedicles to be screwed, and to examine the neighboring tissues that are vulnerable to screw insertion. Acquiring multiple intraoperative X-ray images intending target localization is hazardous to both patients and surgeons. Hence, in the absence of 3D intraoperative imaging devices by utilizing minimal X-ray images, vertebral level identification has been addressed through various computer-assisted tools (CAS).

**Fig. 1:**
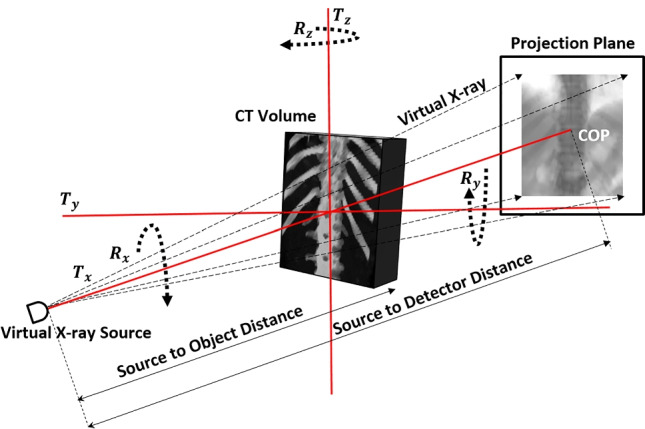
Depiction of DRR generation: The pCT volume exposure to virtual X-rays to generate synthetic X-ray image

Numerous methods had been applied for vertebral level identification and localization utilizing multi-dimensional imaging systems. These approaches can be broadly classified based on the techniques being applied such as machine learning [2–4], segmentation [5], and registration [1, 6]. The proposed method partly incorporates an intrinsic type of intensity-based 3D-2D registration for vertebral level identification in the anterior-posterior (AP) X-ray view. A conventional intensity-based 3D-2D registration system includes digitally reconstructed radiograph (DRR) generation, similarity measurement, and optimization process. DRR generation is a subprocess of intensity-based image registration, wherein a 3D preoperative CT (pCT) volume image is projected onto a 2D projection plane, as depicted in Fig. 1. The DRR-based image registration technique was claimed to be the most accurate image registration system alternative to feature-based image registration systems [7]. DRR-based registration utilizes the entire image intensity details rather than limited details such as points, curves, contours, or surfaces to generate projection images possessing structural correspondence with real X-ray images. Additionally, it was claimed that unlike feature-based registrations, DRR-based registrations are free from registration inaccuracies due to segmentation errors. However, the computational complexity, multiple local maxima, limited capture range, and visual dissimilarities of DRR images from the real X-ray images hamper the applicability of DRR-based image registration [7, 8].

To function as an assistant tool in identifying and estimating the target vertebral pose in intraoperative fluoroscopic images, a three-level registration framework is proposed. The proposed work was targeted for estimating C-arm’s geometrical pose to generate a projection image possessing vertebral structure and pose as that of an intraoperative X-ray image. The process includes an iterative vertebral pose optimization process and the generation of synthetic X-ray images from pCT images. In the framework, similarity-based measurement was performed for the initial pose estimation about the spatial region. In the source to detector (S-D) direction, around the familiar anatomical area, a dimension-wise registration was performed. Finally, to minimize Euclidean distance between reference and projected landmarks, a finer level registration was performed.

The coarse registration was conducted based on the intensity similarity measurements between DRR and X-ray fluoroscopic images. The similarity between two images can be measured by considering the differences in intensities, differences in gradient-related information, and/or shared pixel distribution information. The similarity measure between the DRR and real X-ray image depends on multiple factors such as intensity of images, differences due to quantum noise, distortion and X-ray scattering, X-ray beam hardening, veiling glare, pCT slice resolution, and thickness [8]. In medical image registration, various similarity metrics were applied. Mutual information (MI) and normalized cross-correlation (NCC) are classified to be global intensity correspondence similarity measures. On the other hand, pattern intensity (PI), gradient difference (GD), gradient correlation (GC), and gradient intensity (GI) are categorized as local intensity correspondence similarity measures. According to the study conducted by Markelj et al. [7], local intensity correspondence similarity measures are asserted as more robust than global intensity correspondence similarity measures. Precisely, intensity-based 3D-2D registration majorly depends on the preprocessing of pCT and postprocessing of the projection image, projection techniques, target anatomy, imaging device system configurations, etc.

To match anatomical landmarks between 3D and 2D planes and to overcome issues of intensity-based registration such as capture range and multiple local maxima traps, a variant of ICP-based finer registration is proposed. The ICP-based registration was performed for C-arm’s geometrical pose estimation to map vertebral pose between 3D and 2D images. This type of finer level registration without generating computation-intensive projection images makes the system convenient for faster intraoperative pose estimation. Finally, a high-resolution DRR image was generated at the optimum registration point to visually confirm the estimated ICP-based registred transformation parameters. The developed 3D-2D landmark correspondence-based registration framework can also be aimed for target identification, localization, and pose estimation irrespective of the anatomical region.

## Literature review

The literature review includes the significance of various types of DRR generation techniques, alternatives to optimization-based conventional intensity-based 3D-2D registrations and ICP-based 3D-2D registrations, and the degree of potential of various methodologies utilized in vertebral level identification in X-ray images.

### DRR generation technique applied in 3D-2D registration

The Raycasting-based DRR generation is an accurate way to generate a perspective projection of a 3D CT image, but at a higher computation time $O(n^3)$ [7, 8]. Light field and Fourier slice theorem–based DRR generations were explored to minimize computation time required for the projection image generation. In light field-based DRR generation, a ray of data structure is generated in the preprocessing stage prior to DRR computation. This data structure is a database of rays with attenuation recorded from multiple viewpoints computed prior to intraoperative DRR generation. DRR generation is performed by cumulating pCT attenuation coefficients by eliminating replicated rays reducing DRR computational complexity from $O(n^3)$ to $O(n^2)$ and maintaining an acceptable signal-to-noise ratio closer to the conventional raycasting based DRRs [9]. In the Fourier slice theorem-based approach, the IFFT of the extracted central 2D slice from the 3D spectrum represents the DRR image in a direction perpendicular to the extracted central slice. The method generates orthographic projections reducing DRR computational complexity from $O(n^3)$ to $O(n^2logn)$ [10]. Tetrahedra, shear warp factorization, splatting, and cylindrical harmonics are some of the other alternative techniques developed for DRR generation [11–15].

### Regression-based 3D-2D registration

To minimize dependability from the optimization techniques and to perform faster image registration, a hierarchical regression-based image registration framework was developed [16, 17]. The designed framework provided solution to small capture range issues that are usually encountered during the conventional intensity-based registration process. A CNN-based regression framework was designed to estimate residual transformation parameters by computing local image residuals between DRR and real X-ray images. The design of the 3D-2D registration framework was functionally divided into learning and application stages. In the learning stage, the network was trained to map the relation between transformation parameters and feature differences between the projected and real X-ray images. Subsequently, in the application stage, the extracted features were referred to estimate the transformation parameter. The difficulty level of out-of-plane registration was overcome by dividing the search space into smaller zones and training the individual zones for the image residuals corresponding to hierarchically decomposed transformation parameters. The smaller zones made the regression task simpler but increased the training effort and memory consumption during the runtime. The framework improved registration accuracy through minimal iterations compared to other intensity-based registrations. The experiment was conducted on clinical implants [17] and resulted in moderate registration accuracy in the anatomical registration [16].

### Vertebral level identification in X-ray image

Intensity-based CT to fluoroscopic X-ray image registration systems known as LevelCheck algorithms have been proposed for vertebral level identification in AP and/or lateral X-ray images [1, 6, 18–20]. In a study by Otake et al. [1], preoperative CT vertebral centroids were overlaid on X-ray images using hierarchical registration employing iterative optimization. In a study by Otake et al. [20], 3D-2D registration was conducted in the uncalibrated C-arm system condition and unconstrained source to detector distance condition. The optimization included estimation of transformation parameters in the 9 degree-of-freedom (DOF) search space, which included tuning of additional translational parameters associated with the relative position between the object and X-ray source with respect to the fixed detector. The designed 9DOF registration framework provided better registration accuracy than the 6DOF registration framework at the cost of higher computation time.

Considering various registration scenarios and anatomical deformation, a normalized gradient intensity similarity metric and multi-start CMAES optimizer-based 3D-2D registration framework was designed for vertebral localization in AP X-ray images [6]. The partitioning of search space into subspaces and performing an independent search in these subspaces utilizing the global multi-start strategy guided to overcome false local maxima and short capture range issues. A registration scenario with fewer multistarts, large degrees of deformation, and poor initialization reduced the robustness of the registration system. Specifically, to improve robustness against image content mismatch, various gradient-based similarity metrics have been applied in vertebral centroid registration between CT and lateral plane X-ray images [19]. The registration was performed in the presence and absence of polygonal mask, and different number of multi-starts. The Gradient Orientation similarity metric was found to be robust because it equally weights vertebral gradients and extraneous objects; and hence, a larger vertebral body gradient region in the lateral plane made it convenient to localize robustly under different registration scenarios.

In another variant of the LevelCheck algorithm, a multi-stage registration framework was proposed by Ketcha et al. [21]. The framework performed vertebral level registration under spinal deformation constraints in the thoracolumbar region. To provide better registration accuracy, the individual vertebral levels corresponding to its deformation were piecewise registered in multiple stages without employing any segmentation. It was stated that though the framework performed more evaluations compared to its predecessor algorithms, for faster convergence its execution could be extended for parallel operations across multiple stages. Previously discussed LevelCheck algorithms were architectured based on intensity-based registration and hence are prone to generate thousands of DRR images during the optimization stage. Hence, this makes the process computation-intensive and necessitates a high-end GPU-based rendering or parallel computation platform to support faster intraoperative applications.

To perform fully automatic cervical segmentation in the lateral X-ray image planes, a deep learning-based framework was developed by Al et al. [22]. The framework employed a deep learning approach in spine localization, centroid detection, and shape-aware segmentation. The centroid localization was performed through training a model for Gaussian-wise variable annotated ground-truth centroid positions. Deep segmentation model performance outperformed Active Shape Model (ASM)–based segmentation. In the experiment, poor localization errors resulted due to osteophytes and implants. The framework was aimed for vertebral segmentation and centroid localization rather than vertebral level identification.

A pose-driven deep learning technique and hierarchical segmentation-based approach was proposed for compression fracture grading in the lumbar region [23]. Based on extracted local features, a confidence map was developed from a multi-class predictor to predict the possible center position of individual vertebrae. Vertebral levels of the lumbar region were identified from the confidence map. The framework was claimed to be robust enough for lumbar region identification and segmentation—addressing various challenges and degree of robustness under different constraints such as X-ray images with multiple overlapping shadows of ribs and pelvis, poor X-ray contrast, unclear edge boundaries, and inter-patient variability.

A wider region of spinal lateral images was considered for vertebral landmark localization and radiographic spinopelvic parameter generation in designing a deep learning-based model [24]. The experiment was conducted for spine curvature analysis under various spinal pathological conditions. The cervical and lumbosacral region landmarks were localized optimally. The localization in the mid-thoracic region was moderate due to overlapped regions of scoliotic curves. The thoracolumbar region landmark recognition was found to be challenging for the deep learning-based model due to the occlusions by ribs, implants, and bone cement when viewed in the lateral plane.

A fully automatic framework for initial vertebra poses estimation and identification was proposed by Varnavas et al. [25]. To reduce computational complexity and manual interventions, General Hough Transform (GHT) based initial pose estimation was preferred over 2DOF intensity-based localization. In the preoperative stage, for individual vertebral levels, at variable operating range (6DOF), a database of edge points from the DRR images was built to construct a GHT array. Later in the intraoperative stage, the GHT of the vertebral fluoroscopic image was similarity checked with the previously built GHT array. The framework was sturdy for vertebral detection due to the sensitivity of GHT and the gradient similarity metric for subnormal shape differences.

### Iterative control point–based 3D-2D registration

The ICP-based registration and its variants had been practiced in image registration because of their simplicity and low computational complexity. It iteratively estimates the transformation for the closest distance between two point sets of objects or images. The ICP-based registrations are applied to map 3D shapes [26, 27] and surfaces [28]. The ICP registrations are prominent in non-rigid 3D-2D registrations of vessel structures and arteries for its flexibility under variable degree of deformations [29–31]. ICP-based 3D-2D registration was performed for faster and accurate patient and machine positioning in radiotherapy [32]. Using the Z-buffer algorithm, the problem of information loss in the projection image was addressed through retaining source-to-object distance details. The robustness of a variant of ICP-based registration was tested against intensity-based registration to test its applicability in neurointerventions [33]. Due to erroneous 3D-2D point pairs, the ICP-based registration was found to be marginally reliable when experimented with phantom data as against clinical data. However, the capture range and speed of computation of ICP registration were dominating against its alternative. The higher SNR in digital subtraction angiography (DSA) resulted in clear vessel skeleton-centerline extraction and improved the ICP registration accuracy. The registration accuracy deteriorated when the number of vessels considered for point pairing was increased. In another work, to determine 3D bone kinematics, ICP-based 3D-2D rigid registration was performed between high-speed biplanar videoradiography (HSBV) and MRI-based bone models [34]. The registration precision was not sacrificed when 3D bone model points were randomly downsampled to 10%. In another variant of non-rigid ICP-based 3D-2D registration, 3D femur reconstruction was performed utilizing biplanar radiography [35]. The extracted 2D contours were registered with a 3D template surface model using moving least square deformation metric and by avoiding the requirement of shearing and non-uniform scaling transformations. The proposed work provided better surface reconstruction and asserted that the registration accuracy would improve on adjoining elastic deformation characteristics.

## Method

**Fig. 2:**
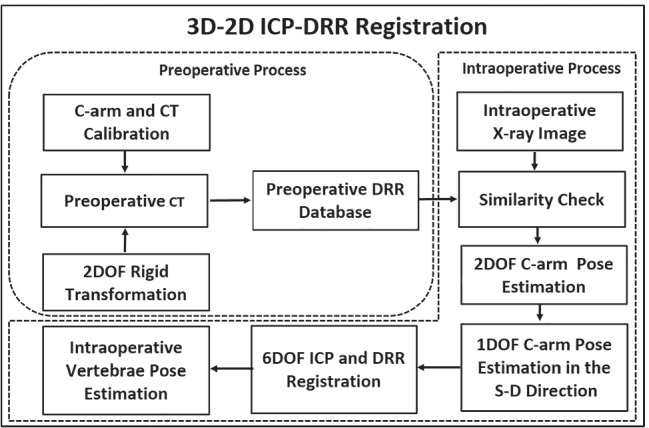
Workflow of 3D-2D ICP-DRR–based registration designed for vertebral level identification and its intraoperative pose estimation

**Fig. 3:**
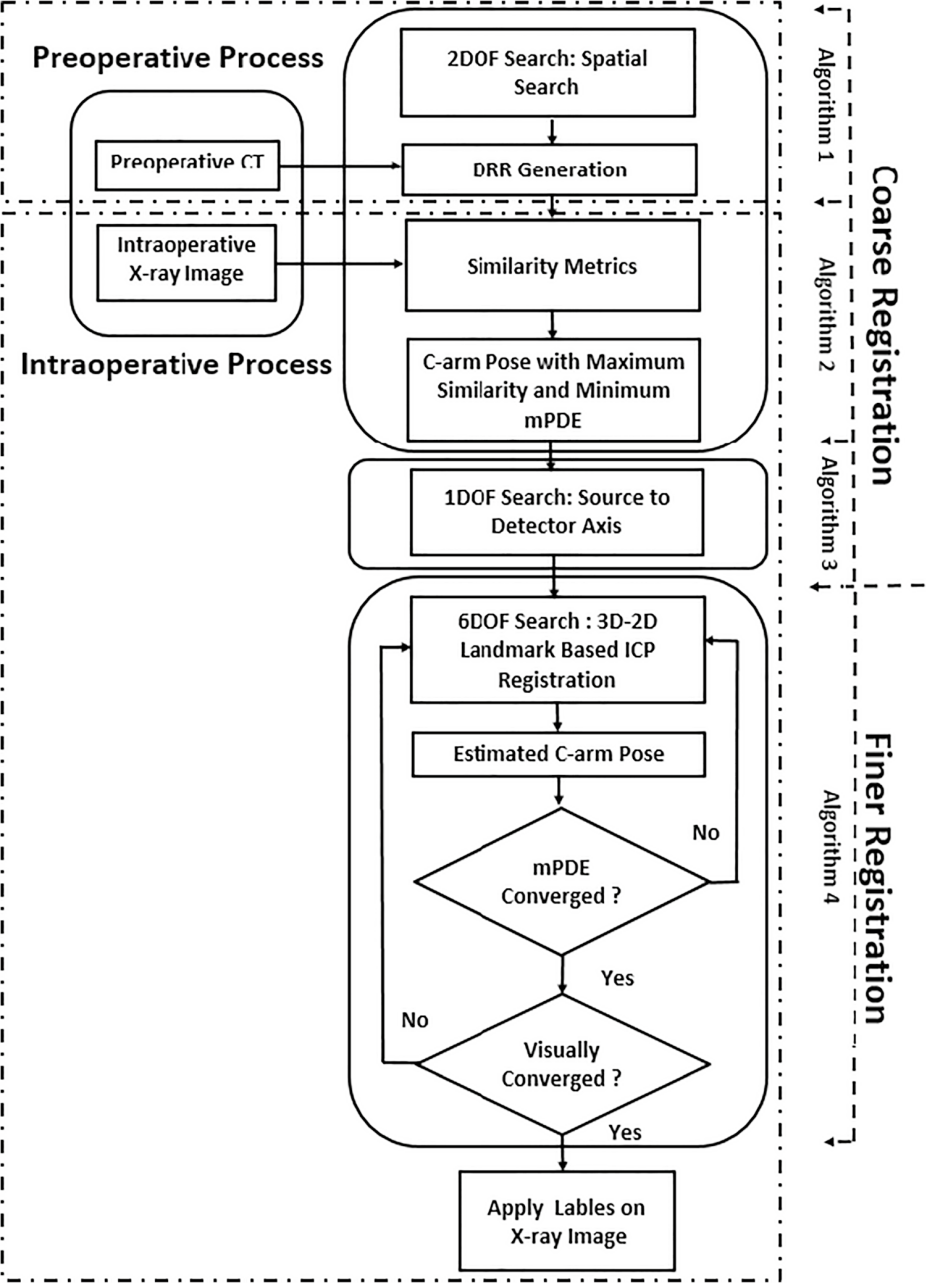
The framework of 3D-2D coarse to finer registration with the demarcation of its subprocesses, algorithms, and modules

The registration framework was targeted for vertebral level identification and its intraoperative pose estimation in an intraoperative environment. A hierarchical registration framework was designed to localize vertebral regions taking the support from an intraoperative X-ray image and multilevel CT data (T5 to L4). The methodology depicted in Fig. 2 incorporates tools and techniques employed in designed registration framework. These tools and techniques were employed during the preoperative and intraoperative processes of the designed image registration system. The preoperative process includes C-arm gantry modeling, and CT and C-arm system calibrations followed by vertebral labeling in CT. The vertebral levels in pCT were marked after knowing the familiar anatomical region and confirming target anatomical landmarks. In the preoperative process, the DRR image database required for the coarse registration was generated (Algorithm 1). The intraoperative process includes similarity measurement between DRR image and X-ray image (Algorithm 2), coarse localization in the S-D direction (Algorithm 3), and ICP-based finer registration for intraoperative vertebral pose estimation (Algorithm 4). A detailed hierarchical framework is depicted in Fig. 3.

### Preoperative process

The preoperative process was defined to conduct faster registration. The process includes C-arm camera modeling, system calibration, vertebral labeling in pCT, and DRR database generation required for intraoperative coarse registration.

#### C-arm pose transformation modeling

The C-arm pose modeling process includes modeling of geometrical transformation of C-arm imaging device for DRR generation at different angles and positions as per the conventional C-arm imaging system. It was suitable to prefer rigid body transformation for our clinical study since the vertebral region is a bony rigid composite and it was assumed that it would not undergo any structural deformation during intraoperative image acquisition. The rigid body registration includes translational ($T_x$, $T_y$, $T_z$) and rotational ($R_x$, $R_y$, $R_z$) transformations known as extrinsic parameters. The projected image also depends on the intrinsic parameters of the imaging device—crucially pixel size ($P_x$, $P_y$) to focal length (SDD) ratio in both horizontal and vertical directions of the projection plane. During the registration process, the center of projection (COP) of the source point on the detector plane guides in identifying the center point of projection. A 2D projection point (u,v) was computed from extrinsic ($T_{extrinsic}$) and intrinsic parameters ($T_{intrinsic}$) of C-arm device as defined in Eq. 1.

1 $$\begin{aligned} (u,v,1)^T= T_{intrinsic} T_{extrinsic} P_{CT} \end{aligned}$$

The factor $T_{intrinsic}$*$T_{extrinsic}$ is the projection matrix that transforms a 3D pCT coordinate point “$P_{CT}$” to the 2D projection plane [1]. During the optimization process, the modeled C-arm position would get tuned iteratively about its initial pose such that mPDE minimizes over successive iterations.

#### DRR generation

In the raycasting process, the attenuation coefficients are weighted by intersection length “*l*” and cumulated along the ray traversing through the pCT volume image as defined in Eq. 2 [8].

2 $$\begin{aligned} I=I_o\exp ^{-\sum \mu (E_{CT})_i*l_i} \end{aligned}$$

Where “*I*” is the projected pixel intensity through tissue volume, “$I_o$” is the X-ray intensity subjected to no attenuation, and “*l*” is the voxel intersection length and “*i*” denotes voxel index [36].

Generated DRR image and real X-ray image always differ because of multiple factors such as X-ray intensity difference between different modalities, pincushion distortion in an X-ray image, X-ray source-detector position relative to patient pose (supine or prone), presence or absence of surgical devices in intraoperative X-ray image, and resolution of CT image dataset. The difference between DRR and real X-ray images can be minimized by preprocessing pCT images and X-ray images through image processing techniques such as scaling the voxels to different intensities, windowing pCT intensities of tissue interest [37], morphological image processing [38], or utilizing postprocessing techniques such as texture extraction and contrast enhancement using histogram equalization [39]. However, despite applying preprocessing and postprocessing techniques, there exist differences between DRR and X-ray images that are usually quantified in terms of SNR and image contrast.

#### Preoperative calibration

The pCT study datasets included spinal columns ranging from the mid-thoracic to lumbar vertebrae (T5 to L4). The axial pCT images were acquired along the craniocaudal axis with the patient being in a supine position utilizing Philips CT system set to peak X-ray generator voltage ranging from 120 to 140 kVp and X-ray tube current ranging from 300 to 400mA. The experiment included datasets specifically acquired for diagnosis purposes and spinal fusion surgeries. During the raycasting process, the anterior-posterior (AP) axis of a pCT imaging system was aligned with the source to detector axis of the C-arm system. The pCT volume center was aligned with the isocenter of the C-arm image coordinate system. Since in pedicle screw insertion surgery, the C-arm’s X-ray source is placed below the operating table, during the raycasting process, the anterior region of the vertebrae was exposed towards the X-ray source [1]. The pixel size and focal length of the C-arm system were acquired from the calibrated C-arm system configuration. The C-arm source and detector were modeled using a transformation matrix for translational ($T_x$, $T_y$, $T_z$) and rotational ($R_x$, $R_y$, $R_z$) movements as depicted in Fig. 1. During the raycasting process, the pCT center and isocenter = (SDD/2, 0, 0), the source position ($x_s$, $y_s$, $z_s$) = (0, 0, 0), and the C-arm position ($T_x$, $T_y$, $T_z$, $\theta _x$, $\theta _y$, $\theta _z$) = (0, 0, 0, 0, 0, 0) were defined as the nominal position to generate synthetic X-ray images along the AP axis.

#### Preoperative CT labeling

The datasets included adolescent patients’ spinal images who had undergone spinal injuries, specifically in the thoracolumbar region. The collected dataset possesses complete axial pCT images, and vertebral levels ranging from T5 to L4 were cropped for the DRR database generation. The dataset specifications of CT and vertebral levels from intraoperative X-ray images considered in our experiment are given in Table 2 of the Annexure section. Since the anatomical transition from T12 to L1 both in the X-ray images and pCT images can be easily identifiable by surgeons, we chose floating ribs or thoracic to lumbar region transition as the landmark for the pose validation during the different stages of registration. The coordinates of the vertebral centroids in pCT were marked such that they attained the center position when viewed in the axial, coronal, and sagittal planes.

#### Preprocessing of CT image

The X-ray photons pass through the patient’s body through different tissues undergoing a different level of attenuation. In CT systems, these attenuation coefficients are represented in the Hounsfield unit (HU) scale where every voxel’s $HU_x$ value is mapped to linear attenuation coefficients ($\mu (E_{CT})$) relative to water attenuation coefficient ($\mu _{water}(E_{CT})$) at X-ray energy $E_{CT}$ as defined in Eq. 3 [1].

3 $$\begin{aligned} \mu (E_{CT})=\frac{1000+HU_x}{1000} \mu _{water}(E_{CT}) \end{aligned}$$

#### Projection image database generation

In the coarse registration procedure, both pCT and X-ray images were downsampled to a resolution of 4mm $\times$ 4mm, and similarity was measured between projected and real X-ray images. The coarse registration requires a DRR database that was generated in the preoperative process. The projection of the pCT image was generated from the raw cropped CT data by perturbing the C-arm position along the superior-inferior (SI) and mediolateral (ML) directions ranging from −50 to +50 mm in step interval of 2 mm. The source to detector distance was fixed such that around the fractured region at least three to four vertebral levels would get displayed on projection. Hence, the initial coarse search database had 2601 DRR images to be similarity checked with real X-ray images. In the case of the lower vertebral study, i.e., in the third dataset, more DRR images were generated along the patient’s superior-inferior axis. A pseudocode algorithm for generating the DRR database in the preoperative process and utilized to perform coarse registration during the intraoperative process is given in Algorithm 1.

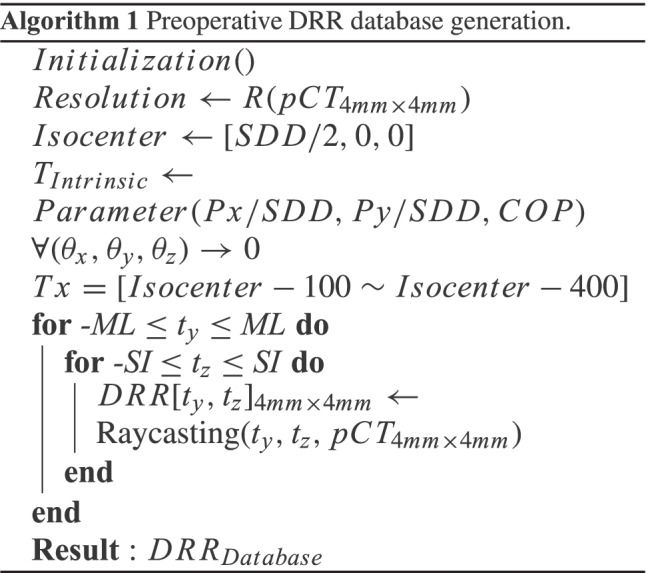



### Intraoperative process

The intraoperative process primarily includes similarity measurements for spatial localization (Algorithm 2), localization in the S-D direction (Algorithm 3), and ICP-based 3D-2D registration (Algorithm 4). The detailed registration framework is shown in Fig. 3. The objective of the designed framework was intensively oriented towards selecting similarity metric which is robust enough to provide multilevel vertebral localization between CT and X-ray datasets when acquired under different X-ray generator settings.

#### Initial pose estimation: spatial search

Similarity measurement was performed using five similarity metrics namely mutual information (MI), gradient information (GI), peak signal to noise ratio (PSNR), structural similarity index metric (SSIM), and normalized cross-correlation (NCC). Across all datasets, the similarity metric which provided better localization in terms of mPDE measurement was considered the best similarity metric for our experiment. The similarity metrics employed during the coarse registration are defined as follows.

Mutual information (MI)- If A(a, b) is a DRR image and B(a, b) is an X-ray image, then the mutual information, entropy, and joint entropy are defined as in the order of Eqs. 4, 5, and 6.

4 $$\begin{aligned} MI(A,B)= & {} H(A)+H(B)-H(A,B)\end{aligned}$$

5 $$\begin{aligned} H(A)= & {} -\sum P_A logP_A\end{aligned}$$

6 $$\begin{aligned} H(A, B)= & {} -\sum \limits _{a, b} P_{A B}(a, b) \log P_{A B}(a, b) \end{aligned}$$

On accurate registration, the joint entropy between the DRR image (A) and X-ray image (B) would be minimum—maximizing individual entropies reflecting the fact that overlapping regions appearing in both of the images are similar.

Gradient intensity (GI) - The gradient intensity as a similarity measure is defined in Eq. 7.

7 $$\begin{aligned} GI(A,B)= & {} \sum \limits _{a,b} w(a,b)min(|\bigtriangledown A(a,b)|,|\bigtriangledown B(a,b)|)\end{aligned}$$

8 $$\begin{aligned} w(a,b)= & {} (cos\alpha _{a,b}+1)/2\end{aligned}$$

9 $$\begin{aligned} cos(\alpha _{a,b})= & {} \frac{ \bigtriangledown A(a,b). \bigtriangledown B(a,b)}{|\bigtriangledown A(a,b)|.|\bigtriangledown B(a,b)|} \end{aligned}$$

Where “*w*(*a*, *b*)” and “$\alpha (a,b)$” are the weighting function and angle between the gradients. The min operator between the magnitude of gradients excludes the extraneous gradients due to the sole presence of surgical tools in intraoperative images; thereby, it is robust against the mismatches between the two images projecting the same anatomy. Additionally, it has been claimed as sturdy against mismatches caused due to soft tissue resection and the energy difference between the imaging modalities [1].

Peak signal to noise ratio (PSNR) - The peak signal to noise ratio was chosen as a similarity measure in image registration [36]. For an image of size M*N, PSNR in dB can be defined as in Eq. 10.

10 $$\begin{aligned} PSNR=10log_{10}\frac{ R^2}{MSE} \end{aligned}$$

Where “*R*” is the peak intensity of an image and “*MSE*” is the mean squared error as defined in Eq. 11.

11 $$\begin{aligned} MSE=\frac{1}{M*N}\sum \limits _{a,b}{ [A(a,b)-B(a,b)]^2} \end{aligned}$$

Structural similarity index metric (SSIM) -  Structural similarity index metric is the similarity metric for quality perception—modeled after the human visual system. It depends on image components such as luminance, contrast, and structure, as described in Eq. 12 [40]. The SSIM measures overall closeness between the images in terms of luminance (L), contrast (C), and structure (M).

12 $$\begin{aligned} {SSIM}(A,B)=L(A,B)C(A,B)M(A,B) \end{aligned}$$

Normalized cross-correlation (NCC) - Normalized cross-correlation measures the correlation coefficient between two images A and B having mean intensities $\bar{A}$ and $\bar{B}$ as defined in Eq. 13.

13 $$\begin{aligned} NCC(A,B)=\frac{\sum _{a,b}(A(a,b)-\bar{A})(B(a,b)-\bar{B})}{\sqrt{\sum _{a,b} (A(a,b)-\bar{A})^2} \sqrt{\sum _{a,b}(B(a,b)-\bar{B})^2}} \end{aligned}$$

A pseudocode for coarse search in the spatial region is given in Algorithm 2. A similarity metric (SM) with maximum similarity value and minimum mPDE was considered the best metric for initial localization ($T_{2DOF}$).

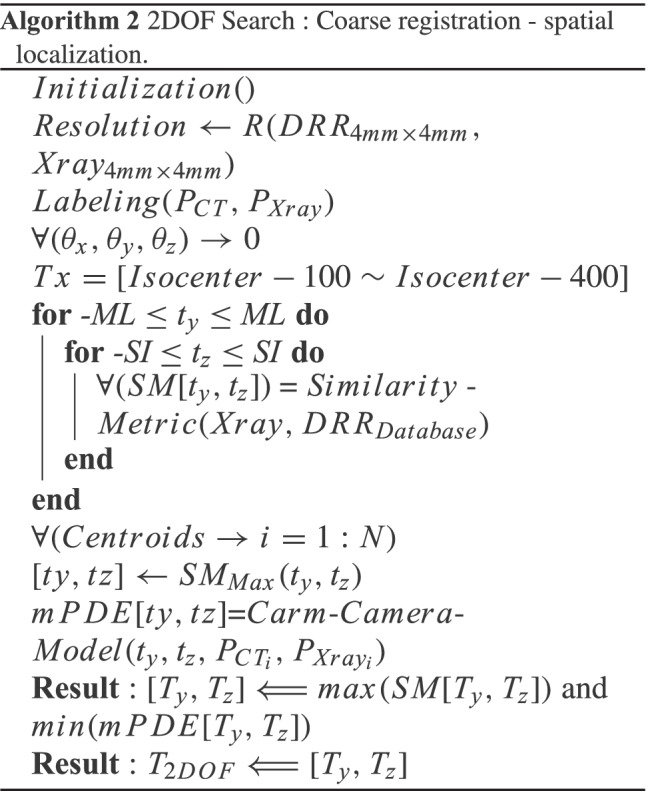



#### Initial pose estimation: source to detector search

**Fig. 4:**
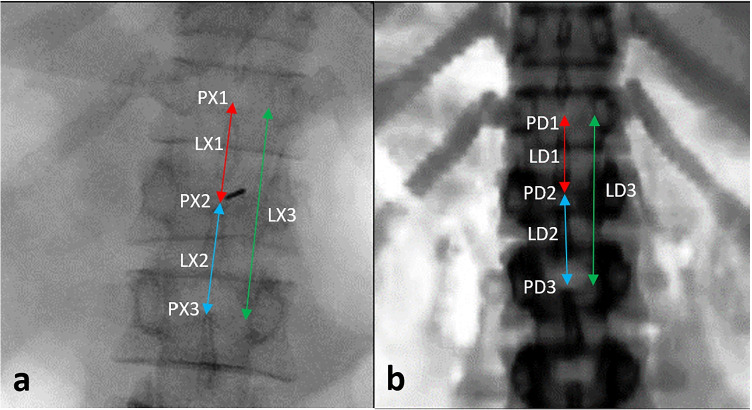
The source to detector direction localization: Intervertebral distance measurement between different vertebral levels in X-ray image (**a**) and DRR image—generated after initial spatial localization (**b**). Source: Orthopaedics and Radiology Department - KMC Manipal

The out-of-plane translational ($T_x$) registration in source-to-detector direction was challenging due to the spatial divergence and uneven similarity pattern. Hence, initial guess in the S-D direction was perceived through the manual registration procedure. Initially, according to the C-arm camera model configuration, the source was placed at the farthest point (FP), i.e., 40 cm or 50cm away from the nominal position. Then, by positioning the X-ray source centering the previous search space parameters ($T_y$ and $T_z$), centroid points were projected from 3D to the 2D projection plane by moving the source towards the CT volume in a step interval of 10 mm. The differences in Euclidean distance between the intervertebral projected centroids and true centroids were measured as depicted in Fig. 4. This procedure points out the approximate magnification factor that is reflected in the acquired X-ray image, and also enhances speed of convergence during the finer registration stage. At the end of this registration, all the three translational parameters($T_x$,$T_y$,$T_z$) get approximately tuned to intraoperative X-ray image pose. After the search in the S-D direction, to visually confirm the initial transformations possessing proximal transformations as that of real X-ray images, the DRR image can be generated at an isotropic resolution of 0.5 mm. A pseudocode algorithm for coarse localization in the S-D direction is defined as in Algorithm 3.

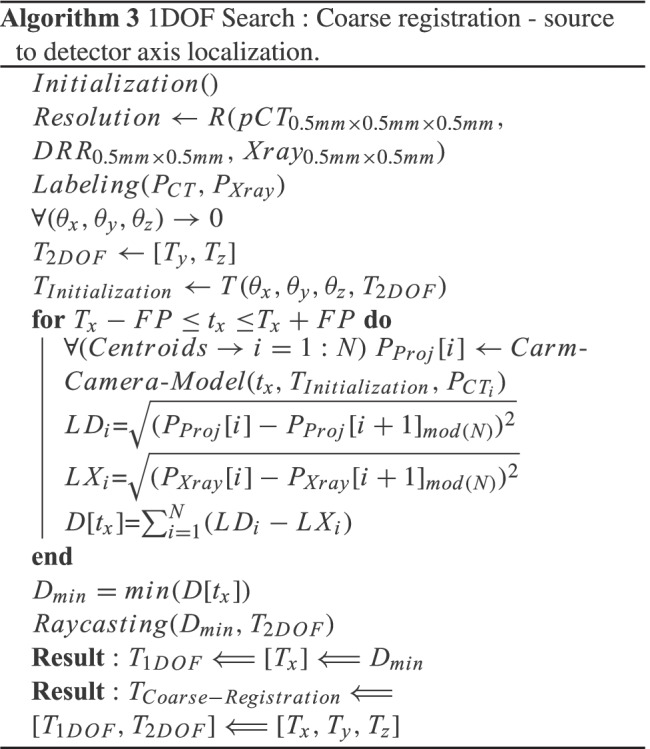



#### Finer registration: 6DOF search

Since, after multiple iterations, intensity-based similarity measurements at higher resolution were not consistently converging towards global maxima, we have employed a novel technique to perform finer registration. The finer registration was performed by minimizing the Euclidean distance measurements between projected centroids and true centroid landmarks. This procedure bypasses the requirement of computation-intensive DRR generation as usually practiced in conventional intensity-based registration. The data coordinate system resolution was defined such that anatomical landmarks can be precisely labeled both in CT and X-ray images.

After initial pose localization, when the vertebral structural appearance in the DRR image falls within the vertebral boundary region as in the X-ray image, finer registration was initiated. Iteratively, the Euclidean distance between projected landmarks and true landmarks was minimized, estimating the C-arm’s transformation for minimum mean projection distance error (mPDE) measurement. Just by the projection of centroid points, we cannot clinically validate the registration framework; hence, on finer registration, we have visually verified the projected image relative to anatomical landmarks that are visible in the target fluoroscopic image. When the final registration position upon initial referenced search space resulted in a minimum mPDE, and when the DRR image at this registration point structurally and orientationally resembled the intraoperative X-ray image, the registration process was terminated.

Covariance matrix adaptation evolution strategy (CMAES) optimization scheme was applied for finer registration, wherein the search space parameters are sampled out from the multivariate normal distribution [41]. These sample populations are characterized after their mean, standard deviation, and covariance matrix, as stated in Eq. 14. In every generation of evolution, the fitness value is computed at all $\lambda$ sample populations, and $\mu$ best sample points called offspring points are selected. The mean and covariance of selected samples are adapted across generations, converging the search space to the optimal bounded region.

If $x^t$=$x_1^t$,$x_2^t$,$x_3^t$,......,$x_n^t$ $\in$ $\mathfrak {R}^n$ denote *n* objective vectors, in every *t* iteration, the objective value “*f*” computed at $x^t$ would get optimized over successive number of generations. The search point distribution can be represented as per Eq. 14 [41].

14 $$\begin{aligned} x_k^{(g+1)}=m^{(g)}+\sigma ^{(g)}N\left( 0,C^{(g)}\right) \sim N\left( m^{(g)},\left( \sigma ^{(g)}\right) ^2,C^{(g)}\right) \end{aligned}$$

Where *k* = 1, 2, ...$\lambda$ are offspring search points and $m^{(g)}$, $\sigma ^{(g)}$, $C^{(g)}$ are the mean, standard deviation and covariance matrix of sample distribution at generation *g* = 0, 1, ...... In every iteration, the fitness value would get optimized to a better level, indicating the fact that the search direction is apparently the same. The fitness function considered during the optimization is defined in Eq. 16. The transformation parameters of the C-arm camera model were estimated through CMAES optimization as given in Algorithm 4 by initializing registration, and loading CT–X-ray landmark point pair positions.

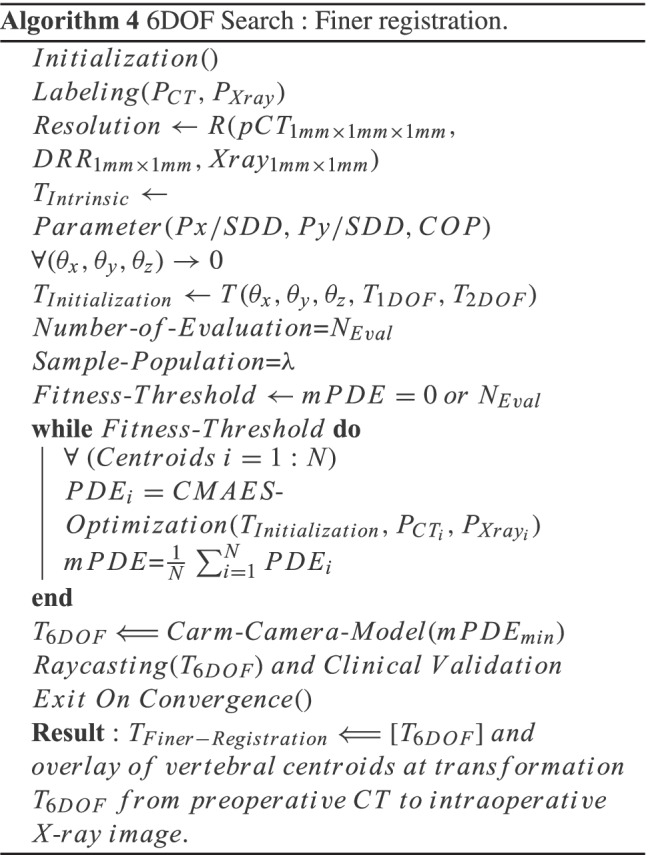



CMAES algorithms are implemented by Hansen in MATLAB [41]. The parameters for step size control and covariance matrix adaptation were defined as mentioned in this work. Population size for sample generation and initial step size for various transformation parameters were tuned based on the initial coarse registration results. For faster convergence, population size can be set to a smaller value, and to avoid local maxima larger population size can be selected [41]. In our optimization process, the sample population and number of evaluations were set to 50 and 2000, respectively. The standard deviation for the transformation in the S-D direction was fixed to 10, and 3 for the rest of the transformation parameters. The mPDE measurement of 0 mm or the number of evaluations was set as the convergence criteria.

## Experiment

The X-ray fluoroscopy images were acquired utilizing Ziehm’s 9-in. C-arm - Image Intensifier system by positioning the patients in the prone position. During pedicle screw insertion surgery, X-ray images were acquired for vertebral level identification. The AP X-ray view was taken as reference image in our registration process. The intraoperative datasets in the experiment were acquired at X-ray generator voltage and X-ray tube current falling in the range of 66 to 86kV and 3.3 to 3.8 mA, respectively. For clear vertebral structural visibility, these settings were tuned according to the patient’s morphology and bone mineral density. In our experiment, target localization was performed on real X-ray images acquired for four patients. From intraoperative X-ray images, a set of three vertebral columns ranging from T11 to L3 was considered for the study purpose. The vertebral levels specific to a dataset, its pCT data resolution, and voxel dimensions are given in Annexure - Table 2. Though the intraoperative images were acquired without any C-arm gantry rotation, during the finer registration stage, the C-arm model position was perturbed even rotationally to compensate the patient positioning differences between preoperative and intraoperative environments. The distances between the projected centroids and X-ray centroids were minimized throughout the optimization process, converging the corresponding anatomical landmarks and vertebral poses for an optimal match. The framework was implemented on the MATLAB platform under Windows 7 Professional 64-bit and HP Z230 workstation configurations.

## Evaluation method

More often, in registration procedures, the fiducial markers and/or external trackers are employed for the patient or the C-arm pose estimation [42]. For ground truth registration, fiducial markers are placed in the study model, and spatial information is tracked using an optical or electromagnetic tracker. Through the registration algorithm, the fiducial marker position is estimated and compared with the transformation matrix derived from the intrinsic and extrinsic matrices of the C-arm imaging system. Finally, the difference between these measures, known as target registration error (TRE), is measured as described below. If $T_{Reg}$ is the transformation matrix derived from the registration algorithm and $T_{GT}$ is the transformation matrix derived after ground truth registration, then mean TRE for “k” pCT coordinate points “$P_{CT}$” is defined as in Eq. 15 [43, 44].

15 $$\begin{aligned} mTRE(P_{CT},T_{GT},T_{Reg})=\frac{1}{k}\sum \limits _{i=1}^{k}T_{GT}P_{CT_i}-T_{Reg}P_{CT_i} \end{aligned}$$

In 3D-2D registration, based on the plane of error measurement, registration error measurements are classified into three types, i.e., TRE, projection distance error (PDE), and reprojection distance error (RPDE) [44]. The registration error TRE is suitable in image-guided navigation systems, wherein localization is performed in 3D by relatively mapping landmarks from 2D image planes. In 3D-2D registration, PDE is a distance metric between estimated and target points measured on 2D projection plane. The RPDE is the distance between two points in 3D obtained after back-projecting 2D test points at estimated projections and 3D gold standard position of it. To validate the design of registration systems, a set of “k” test points are chosen, and aforementioned applicable registration error types are measured.

We measured mPDE to evaluate the designed registration framework by considering vertebral centroid landmarks as reference [1, 45]. The Euclidean distance (ED) was measured between a set of three projected centroids and corresponding X-ray centroids as defined in Eq. 16 [1].

16 $$\begin{aligned} mPDE(P_{CT},T_{Reg},T_{Xray})=\frac{1}{N}\sum _{k=1}^{k=N}T_{Reg}P_{CT_k}-P_{Xray_k} \end{aligned}$$

**Fig. 5:**
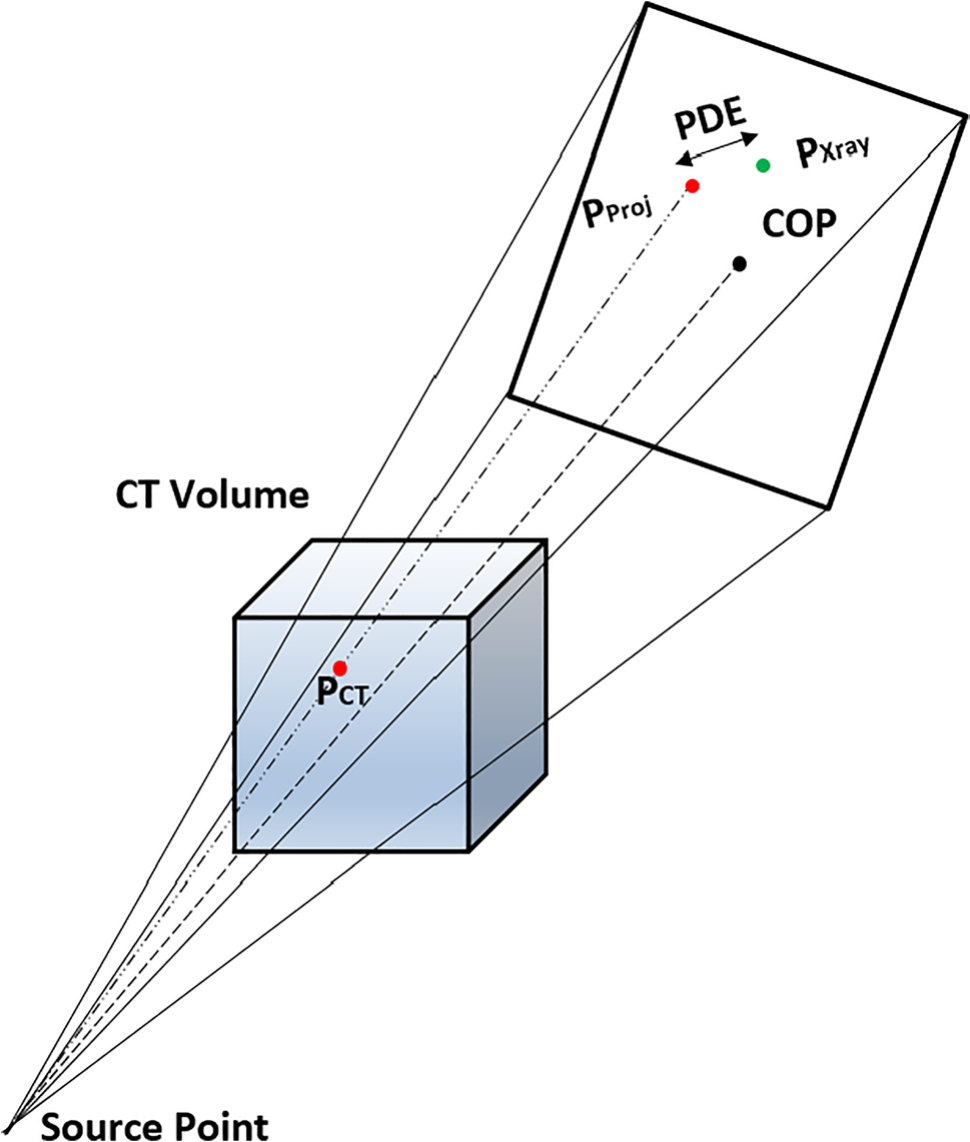
Depiction of C-arm camera model projecting pCT coordinate point and measurement of PDE

Where $T_{Reg}$ is the estimated final registration transformation parameter, “$P_{CT}$” and “$P_{Xray}$” are the vectors of “N” centroid points in the world coordinate system. Figure 5 depicts the projection of the pCT coordinate point on the detector plane and measurement of PDE. Since the framework functions without any marker or tracker, for validation, the optimum C-arm pose was confirmed when the distance between the projected points and target points is minimum. In support, we have verified the final pose by generating a high-resolution DRR image possessing an optimal match with the real X-ray image in terms of shape, structure, and pose. In addition, we validated the framework’s registration accuracy and pose estimation capabilities based on three surgeons’ feedbacks.

## Result

### Coarse registration: intensity-based similarity measurement

**Fig. 6:**
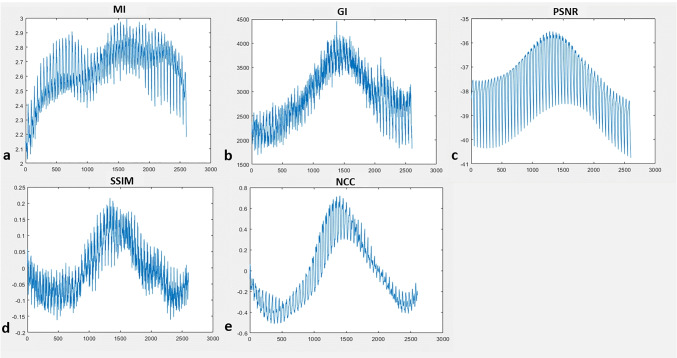
Similarity measurement plot between X-ray image and coarse registration database using MI (**a**), GI (**b**), PSNR (**c**), SSIM (**d**), and NCC (**e**)

**Fig. 7:**
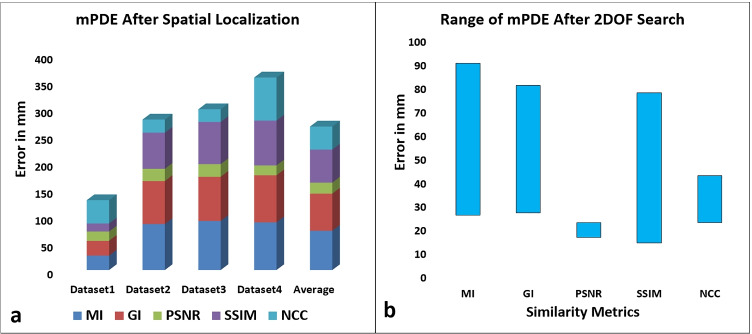
Stacked bar graph of mPDE for various similarity metrics across different datasets (**a**) and range of mPDE for the study dataset after the spatial localization (**b**)

The similarity measurement using different similarity metrics during coarse registration is shown in Fig. 6. It was observed that the PSNR metric consistently provided a better initial point of localization across all the datasets. The variation in PSNR-based similarity measure was distributed almost evenly about coarse registration point (Fig. 6c). The average mPDE for the study dataset after PSNR-based coarse registration was found to be 20.41 mm. In Fig. 7, mPDE and its range after spatial localization are displayed as per the different similarities and the study datasets.

### Coarse registration: intervertebral distance–based localization in the S-D direction

**Fig. 8:**
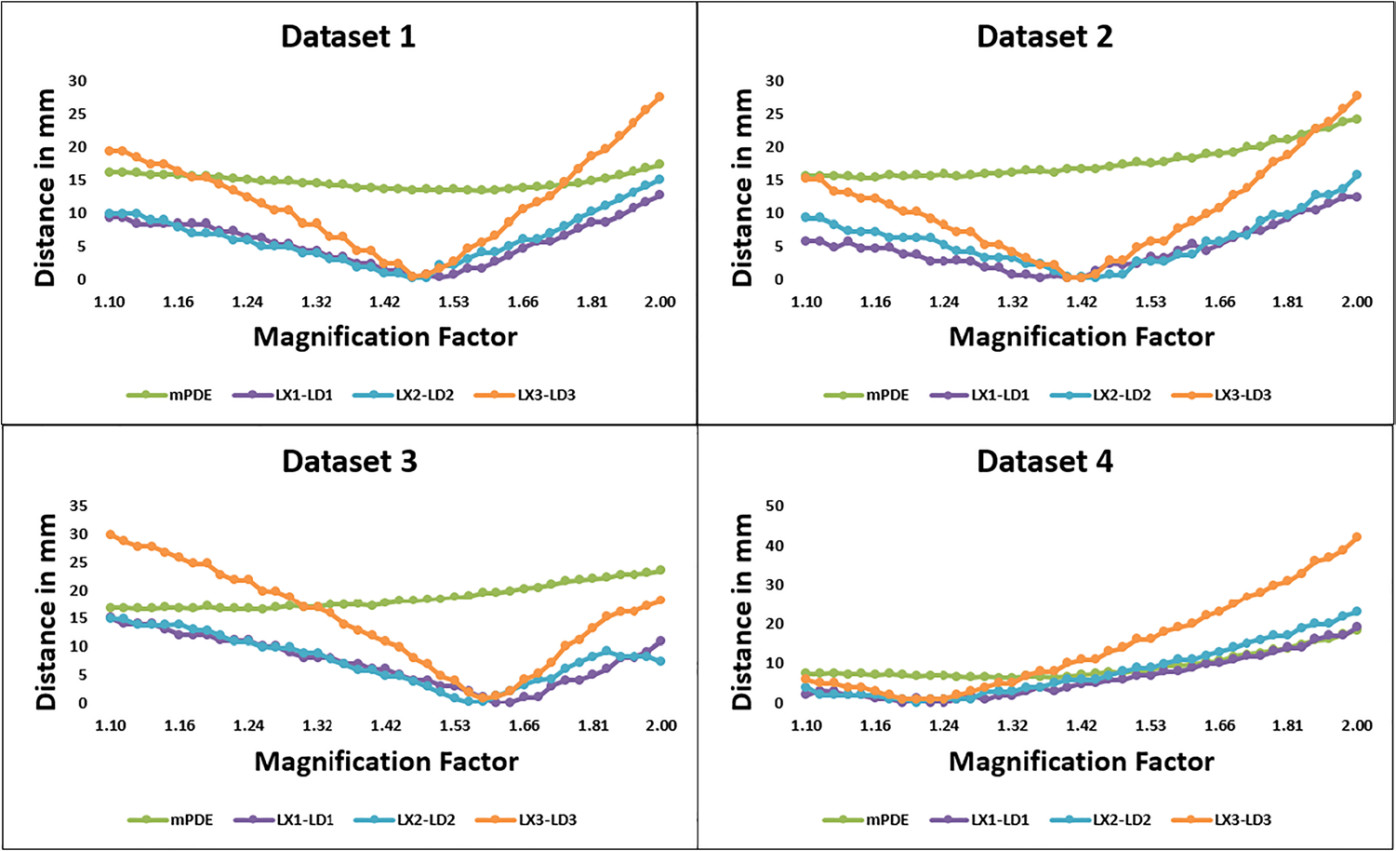
Intervertebral length differences between DRR image and X-ray image and mPDE measurements during the search in the source to detector direction are plotted against the magnification factor (LX and LD labels are as depicted in Fig. 4)

In the S-D direction, an approximate localization point was achieved when the metric converged towards a minimum value. From Fig. 8, it can be observed that the magnification factor of the study dataset varies in a range of 1.2 to 1.6, indicating an actual magnification factor as that of the intraoperative C-arm imaging environment projecting four to five vertebral levels.

### Finer registration: ICP registration

**Fig. 9:**
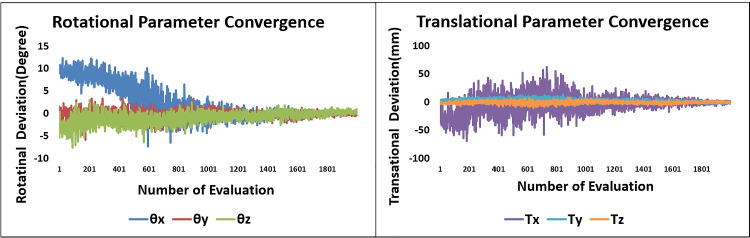
Transformation parameter convergence about the final estimation over successive number of evaluations (dataset 1)

**Fig. 10:**
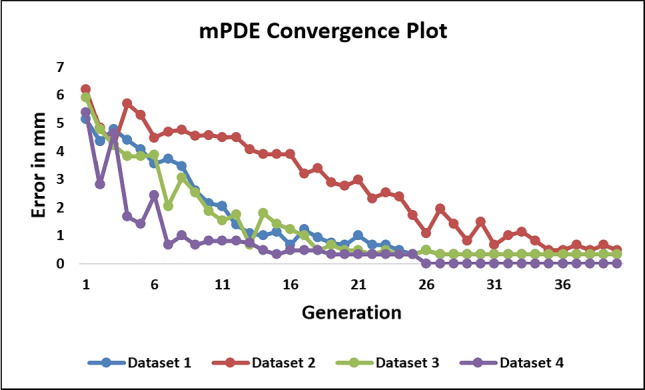
Convergence of mPDE measurement over successive generations in different datasets

**Fig. 11:**
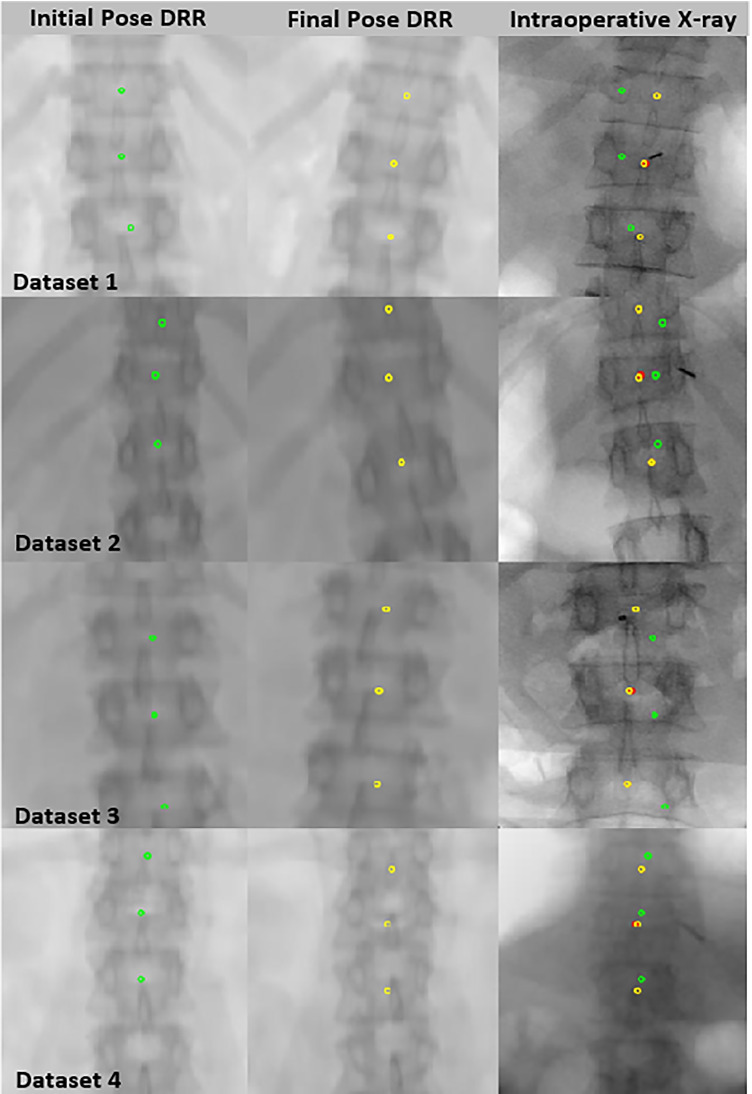
Overlay of vertebral centroids from pCT image on DRR image at initial pose (column 1, green), final pose (column 2, yellow), and on intraoperative X-ray image (column 3, red)

The convergence of transformation parameters towards optimum transformation across a successive number of iterations is shown in Fig. 9. The mPDE convergence plot after initial pose estimation is shown in Fig. 10. The initial mPDE measurements were due to the spinal rotation, displacement, and deformation that occur due to the positioning differences between the preoperative and intraoperative imaging environments. The mPDE measurement at the initial and the final estimated poses for various datasets is displayed in Table 1. The mPDE after the first generation of optimization was in a range of 5 to 6.5mm and progressively converges up until the final generation registering vertebral pose optimally, as displayed in Fig. 11. The pCT centroid landmarks were registered optimally, projecting the corresponding anatomical region in and around the target region. However, there were challenges associated with the tuning of out-of-plane transformations that can be addressed by individually registering vertebral levels utilizing 3D-2D landmarks from multiple planes.

**Table 1:** The mPDE measurement for different datasets at the initial and final points of registration

Dataset	Initial mPDE	Final mPDE
1	13.62	0.33
2	09.36	0.47
3	18.85	0.33
4	06.88	0.33

## Discussion

The necessity of various types of preprocessing and postprocessing techniques at the DRR generation stage, DRR computational complexities, and low registration accuracy due to poorly contrasted intraoperative X-ray images were overcome through our designed registration framework. The registration framework referred to anatomical landmarks without utilizing fiducial markers or trackers, hence making the experimental procedure convenient and amicable for vertebral identification and its pose estimation during spinal fusion procedures. The designed framework has the potential to explore pose estimation problems irrespective of anatomy in intraoperative applications through 3D-2D landmark correspondence knowledge and limited generation of projection images. The huge dataset requirement and generalizability issue of machine learning-based registration and multiple local maxima issues of intensity-based registration were overcome through our designed registration system.

The preoperative DRR database creation benefited in terms of time complexity, which is an essential factor for the registration framework to support intraoperative applications. Additionally, at the coarse registration stage, during the DRR database generation, the skull, cervical, prior thoracic levels, and pelvis regions were excluded from the preoperative CT for faster intraoperative registration and to avoid multiple false maxima. Furthermore, to improve initial registration accuracy of coarse registration, horizontally wider intraoperative images possessing vertebral columns along with the attached ribs were considered. A combined metric of maximum intensity-based similarity and minimum projection distance error was considered for spatial region localization. In coarse registration of unpreprocessed datasets, among the considered similarity measures, the PSNR metric was found to be robust. The PSNR metric managed to distinguish vertebrae along the superior-inferior axis despite poor X-ray image contrast and vertebral level structural repeatability. At low resolution, the absence of the rib region in the thoracolumbar region was easily detected by this metric. It was also observed that the PSNR metric provided a spatially closer localization point when the DRR database was created for a translational step interval of 2 mm as against 10 mm. In the case of the fourth dataset, the mPDE measurement was consistently poor across all the metrics except PSNR, crucially due to the blurred edges of the pedicle and vertebral body endplates.

To ease our registration process and for faster finer registration convergence rate, intervertebral length metric-based registration was performed in the S-D direction. Since, in the S-D direction, the cost function was sensitive to the magnification factor, we can bypass manual search to improve the speed of computation. Moreover, feature-based localization in the S-D direction like ours beats the major issue of intensity-based out-of-plane translational parameter registration [25].

Across all the experiments on the studied datasets, on repeatedly performing finer registration about the initial localization, all the estimated final transformation parameters did not get deviated substantially about their mean values. This signifies that the framework is efficient enough for pose reproducibility. This was possible because in its first generation the CMAES optimizer successfully managed to sink all the transformation parameters within a narrow basin. The localization in dataset 2 took higher number of evaluations to depreciate the mPDE value due to the increased degree of noncollinearity between the three vertebral levels and initialization to lower magnification factor. Additionally, in the inter-imaging environmental conditions, variable out-of-plane rotations among different vertebral levels resulted in lower registration accuracy.

The out-of-plane transformation parameters can be estimated more accurately by considering multiple landmarks from the individual vertebrae and their 3D-2D plane landmark correspondence knowledge. On final registration, we generated visually optimal projection images at minimum projection distance error. These projection images visually differ from real X-ray images due to the rigid registration practice of all three levels. However, the optimality of projection images can be further enhanced by registering the individual vertebral levels which are subjected to variable degrees of inter-imaging environmental deformations. Registration accuracy may differ by a length of fewer than 2 mm due to landmark marking variations in both 3D and 2D planes. Furthermore, automatic centroid localization in pCT and X-ray image may reduce the manual interventions.

## Conclusion

The difficulty of vertebral centroid identification and its pose estimation in low-contrast anterior-posterior view X-ray fluoroscopy images was overcome through the process known as preoperative CT to intraoperative X-ray image registration. The PSNR-based similarity metric provided a better initial localization point considering unprocessed data. The ICP-based finer 3D-2D registration facilitated bypassing the computation-intensive DRR generation during the optimization stage, permitting our framework’s applicability in the intraoperative applications. The designed framework accurately registered vertebral levels in the intraoperative application with an average mPDE of 0.36mm within a mean time of fewer than 2 min. The PDE measurement and speed of computation in our process of vertebral level identification and pose estimation were well within the clinical acceptance range. The designed framework could be extended to investigate spinal deformation between preoperative and intraoperative images through individual vertebral registration. Though the framework was truly designed for identifying and localizing vertebral centroids in intraoperative images, it can also be extended to estimate various anatomical poses between preoperative and intraoperative imaging environments.

## Annexure

### Dataset specification

The specifications of the pCT images and intraoperative X-ray images that are acquired for the study purposes are given in Table 2.

**Table 2:** Experimental dataset specifications

Dataset	pCT volume	pCT voxel size (mm)	Intraoperative X-ray vertebral levels
1	$512\times 512 \times 243$	$0.56\times 0.56 \times 3$	T12 to L2
2	$512\times 512 \times 242$	$0.47\times 0.47\times 3$	T11 to L1
3	$512\times 512 \times 244$	$0.44\times 0.44 \times 3$	L1 to L3
4	$512\times 512 \times 211$	$0.58\times 0.58 \times 3$	T12 to L2
